# On Unrooted and Root-Uncertain Variants of Several Well-Known Phylogenetic Network Problems

**DOI:** 10.1007/s00453-017-0366-5

**Published:** 2017-08-22

**Authors:** Leo van Iersel, Steven Kelk, Georgios Stamoulis, Leen Stougie, Olivier Boes

**Affiliations:** 1 Delft Institute of Applied Mathematics, Delft University of Technology, Delft, The Netherlands; 2 Department of Data Science and Knowledge Engineering (DKE), Maastricht University, Maastricht, The Netherlands; 3 CWI, INRIA-Erable and Department of Econometrics and Operations Research, Vrije Universiteit, Amsterdam, The Netherlands

**Keywords:** Binary trees, Fixed parameter tractability, Kernelization, APX-hardness, NP-completeness, Phylogenetic networks

## Abstract

The hybridization number problem requires us to embed a set of binary rooted phylogenetic trees into a binary rooted phylogenetic network such that the number of nodes with indegree two is minimized. However, from a biological point of view accurately inferring the root location in a phylogenetic tree is notoriously difficult and poor root placement can artificially inflate the hybridization number. To this end we study a number of relaxed variants of this problem. We start by showing that the fundamental problem of determining whether an *unrooted* phylogenetic network displays (i.e. embeds) an *unrooted* phylogenetic tree, is NP-hard. On the positive side we show that this problem is FPT in reticulation number. In the rooted case the corresponding FPT result is trivial, but here we require more subtle argumentation. Next we show that the hybridization number problem for unrooted networks (when given two unrooted trees) is equivalent to the problem of computing the tree bisection and reconnect distance of the two unrooted trees. In the third part of the paper we consider the “root uncertain” variant of hybridization number. Here we are free to choose the root location in each of a set of unrooted input trees such that the hybridization number of the resulting rooted trees is minimized. On the negative side we show that this problem is APX-hard. On the positive side, we show that the problem is FPT in the hybridization number, via kernelization, for any number of input trees.

## Introduction

Within the field of phylogenetics the evolutionary history of a set of contemporary species *X*, known as *taxa*, is usually modelled as a tree where the leaves are bijectively labelled by *X*. One of the central challenges in phylogenetics is to accurately infer this history given only measurements on *X* (e.g. one string of DNA per species in *X*) and to this end many different optimality criteria have been proposed [12, 29]. One issue is that algorithms which construct evolutionary trees (henceforth: phylogenetic trees) usually produce *unrooted* phylogenetic trees as output i.e. trees in which the direction of evolution is not specified and thus the notion of “common ancestor” is not well-defined. Nevertheless, biologists are primarily interested in *rooted* trees [23], where the root, and thus the direction of evolution, is specified. In practice this problem is often addressed by solving the tree-inference and root-inference problem simultaneously, using a so-called “outgroup” [27]. However, this process is prone to error (see [38] for a recent case-study) and disputes over rooting location are prominent in the literature (see e.g. [11]).

Moreover, in recent years there has been growing interest in algorithms that construct rooted phylogenetic *networks* [17], essentially the generalization of rooted phylogenetic trees to rooted directed acyclic graphs. One popular methodology is to construct phylogenetic networks by merging sets of trees according to some optimality criterion [18, 24]. For example, in the Hybridization Number (HN) problem we are given a set of rooted phylogenetic trees as input and we are required to topologically embed them into a network $N=(V,E)$ such that the reticulation number $r(N) = |E| - (|V|-1)$ is minimized; the minimum value thus obtained is known as the hybridization number of the input trees. This problem is NP-hard and APX-hard [6] and has similar (in)approximability properties to the classical problem Directed Feedback Vertex Set (DFVS) [22], which is not known to be in APX i.e., it is not known whether it permits constant-factor polynomial-time approximation algorithm. We remind the reader that in the DFVS problem we are given a directed graph $G=(V,E)$ and a positive integer *k*, and we are asked if there is a subset of vertices $S \subseteq V$, $|S| \le k$, such that if we delete the set *S* from *G*, the remaining graph is cycle-free. On a more positive note, there has been considerable progress on developing fixed parameter tractable (FPT) algorithms for HN. Informally, these are algorithms which solve an input instance *x* of HN in time $O( f(k) \cdot \text {poly}(|x|) )$ where here by |*x*| we denote the size of the input instance *x*, *k* is the hybridization number of the input trees and *f* is some computable function that only depends on *k*. FPT algorithms have the potential to run quickly for large |*x*|, as long as *k* is small (see [10] for an introduction), and they can be highly effective in applied phylogenetics (see e.g. [14, 30, 37]). In [5] it was proven that HN is FPT (in the hybridization number) for two input trees and in recent years the result has been generalized in a number of directions (see [31] and the references therein for a recent overview).

One modelling issue with HN is that a poor and/or inconsistent choice of the root location in the input trees can artificially inflate the hybridization number, and this in turn can (alongside other methodological errors) be misinterpreted as evidence that reticulate evolutionary phenomena such as horizontal gene transfer are abundant [23, 34]. To take a simple example, consider two identical unrooted trees on a set *X* of *n* taxa which *should*, in principle, be rooted in the same place, so the hybridization number should be 0. If, however, they are rooted in different places due to methodological error, the hybridization number will be at least 1, and in the worst case can rise to $n-2$. The effect is reinforced as the number of trees in the input increases.

To this end, in this article we study a number of variations of HN (and related decision problems) in which the root has a relaxed role, or no role whatsoever. The first major part of the article is Sect. 3 in which we analyse the Unrooted Tree Containment (UTC) problem. This is simply the problem of determining whether a given unrooted phylogenetic network *N* has a given unrooted phylogenetic tree *T* topologically embedded within it. (Following [13], an *unrooted phylogenetic network* is simply a connected, undirected graph where every internal, i.e. non-leaf, node has degree 3 and the leaves are, as usual, bijectively labelled by *X*). The rooted version of this problem has received extensive interest [7, 15, 33] and, although NP-hard [20], permits a trivial FPT algorithm, parameterized by the reticulation number of *N*. Here we show that UTC is also NP-hard, addressing a number of technicalities that do not emerge in the rooted case, and FPT in the reticulation number of *N*. However, here the FPT algorithm is not trivial. We describe a linear kernel based on contracting common chains and subtrees, and a bounded-search branching algorithm with running time $O(4^k |V(N)|^2)$, where *k* is the reticulation number and |*V*(*N*)| is the number of nodes in the network.

**Fig. 1:**
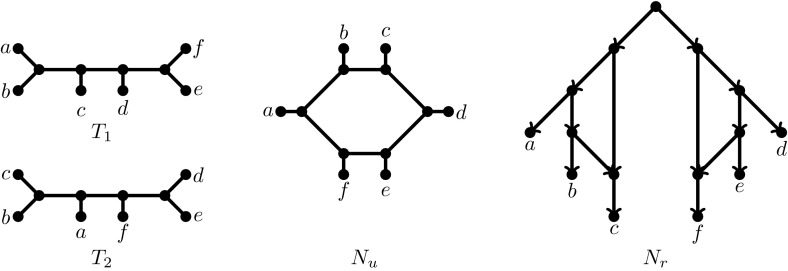
Two unrooted trees $T_1,T_2$, an unrooted network $N_u$ with reticulation number 1 that displays $T_1$ and $T_2$ and a rooted network $N_r$ that displays rootings of $T_1$ and $T_2$ and has reticulation number 2. $N_u$ is an optimal solution to the UHN problem, while $N_r$ is an optimal solution to the RUHN problem

In Sect. 4, a comparatively short section, we consider the Unrooted Hybridization Number (UHN) problem, where both the input trees and the output network are *unrooted*. In this section we restrict our attention to the case when the input has exactly two trees $T_1$ and $T_2$ and we simply ask to find an unrooted network that displays them both such that the reticulation number of the network is minimized. Consider for example the case of Fig. 1 where we are given two *unrooted* trees $T_1, T_2$ as input. $N_u$ is a network that displays them both such that $r(N_u) = 1$ and this is optimal. Slightly surprisingly we show that for UHN the minimum reticulation number of any network that contains both $T_1$ and $T_2$, is equal to the Tree Bisection and Reconnection (TBR) distance of $T_1$ and $T_2$, which in turn, as is well-known, is equal to the size of an optimum solution to the Maximum Agreement Forest (MAF) problem, minus 1 [1]. Hence, the UHN problem on two trees immediately inherits both negative and positive results about TBR/MAF: NP-hardness on one hand, but constant-factor polynomial-time approximation algorithms and FPT algorithms on the other. This shows that, from an approximation perspective, UHN might be strictly easier than its rooted counterpart which, as mentioned earlier, might not be in APX at all. It also means that UHN benefits from ongoing, intensive research into MAF [4, 8, 9, 35].

In the second major part of the article, Sect. 5, we consider the Root Uncertain Hybridization Number (RUHN) problem. Here the input is a set of *unrooted* binary trees and we are to choose the root location of each tree, such that the reticulation number is minimized. See again Fig. 1. In contrast with UHN, if we have to root each of $T_1,T_2$ then the minimum reticulation number is 2 and this is achieved by the rooted network $N_r$. This simple example also shows that UHN can be strictly smaller than RUHN, a point we will elaborate on in the preliminaries. Biologically speaking, RUHN is the most relevant problem we study because it explicitly acknowledges the fact that the input unrooted trees need to be rooted in some way. This highlights the fact that a root exists, but its location is uncertain and we would like to infer the root locations such that the reticulation number of a network that displays them all is minimized. On the negative side we show that this problem, which was explored experimentally in [37], is already NP-hard and APX-hard for two trees. On the positive side, we show that the problem is FPT (in the hybridization number) for any number of trees, giving a quadratic-sized kernel and discussing how an exponential-time algorithm can be obtained for solving the kernel. Similar ideas were introduced for the rooted variant in [32]. Finally, in Sect. 6 we conclude with a number of open questions and future research directions.

We begin with a section dedicated to preliminaries in which we formally define all the models studied in this paper and briefly discuss their differences.

## Preliminaries

A *rooted binary phylogenetic network* $N=(V,E)$ on a set of leaf-labels (also known as *taxa*) *X*, (where $|X| \ge 2$), is a directed acyclic graph (DAG) in which the leaves (nodes with indegree 1 and outdegree 0) are bijectively labelled by *X*, there is exactly one *root* (a node with indegree 0 and outdegree 2), and all other nodes are either *tree* nodes (indegree 1, outdegree 2) or *reticulation nodes* (indegree 2, outdegree 1). As mentioned in the introduction, the *reticulation number* *r*(*N*) of *N* is defined as $|E|-(|V|-1)$, which is equal to the number of reticulation nodes in *N*. In other words, the reticulation number of a network is the number of edges we need to delete in order for the underlying undirected graph to be acyclic (i.e., a spanning tree). A rooted binary phylogenetic network *N* which has $r(N)=0$ is simply called a *rooted binary phylogenetic tree*. Two rooted binary phylogenetic networks $N_1$ and $N_2$ on *X* are said to be isomorphic if there exists an isomorphism between $N_1$ and $N_2$ which is the identity on *X*.

Similarly, an *unrooted binary phylogenetic network* on *X*, where $|X|\ge 2$, is simply a connected, undirected graph $N=(V,E)$ with |*X*| nodes of degree 1 (i.e., leaves), labelled bijectively by *X*, and all other internal nodes, if any, are of degree 3. Although notions of indegree and outdegree do not apply here, reticulation number can still naturally be defined as $r(N) = |E|-(|V|-1)$. An unrooted binary phylogenetic tree is an unrooted binary phylogenetic network with $r(N)=0$. See Fig. 1 for examples of rooted and unrooted networks.

We note that another way to define rooted networks is the following: Let *N* be an *unrooted* network. We select an edge *e* of *N* and we subdivide it. Let this new vertex be the root. Thus we can have at most |*E*(*N*)| many root locations. Each such root location defines a direction of evolution “away” from the root but, due to cycles in the network, many different orientations on its edges are possible, and thus many different rooted phylogenetic networks can be obtained. In fact, as was discussed in detail in [13], for each root location we can have *exponentially* many induced rooted phylogenetic networks.

Throughout the article we will occasionally refer to *caterpillars*. For $n \ge 4$ an *unrooted caterpillar* $(x_1, \ldots , x_n)$ is the unrooted binary phylogenetic tree constructed as follows: it consists of a central path $(p_2, \ldots , p_{n-1})$ with a single taxon $x_i$ adjacent to each $p_i$ (for $3 \le i \le n-2$), two taxa $x_1$ and $x_2$ adjacent to $p_2$ and two taxa $x_{n-1}$ and $x_n$ adjacent to $p_{n-1}$. The two trees shown in Fig. 1 are both unrooted caterpillars with $n=6$. A *rooted caterpillar* is obtained by subdividing the edge $\{p_2,x_1\}$, taking the newly created node $p_1$ as the root and directing all edges away from it.

We say that a rooted binary phylogenetic network *N* on *X* *displays* a rooted binary phylogenetic tree *T* on *X* if *T* can be obtained from a subtree $T'$ of *N* by suppressing nodes with indegree 1 and outdegree 1. Similarly, an unrooted binary phylogenetic network *N* on *X* *displays* an unrooted binary phylogenetic tree *T* on *X* if *T* can be obtained from a subtree $T'$ of *N* by suppressing nodes of degree 2. In both cases we say that $T'$ is an *image* of *T*.

Consider the following problem, which has been studied extensively, and its unrooted variant.

- **Problem:** Hybridization Number (HN)
- **Input:** A set $\mathcal {T}$ of rooted binary phylogenetic trees on the same set of taxa *X*.
- **Output:** A rooted binary phylogenetic network *N* on *X* such that, for each $T \in \mathcal {T}$, *N* displays *T*.
- **Goal:** Minimize *r*(*N*).

The minimum value of *r*(*N*) thus obtained is denoted by $h^{r}(\mathcal {T})$ and this is also called the *hybridization number* of $\mathcal {T}$. Note that in order to verify feasibility of proposed solutions to HN we need certificates that certify that a solution is indeed feasible: such certificates in fact verify that all of the input trees are actually displayed by the network *N* and they are simply the images of each tree in the network. The reason that we explicitly need such certificates is the fact that determining whether a given rooted network displays a given rooted tree is an NP-hard task [20]. This is precisely the Tree Containment problem, defined in more detailed later.

HN is APX-hard (and thus NP-hard) [6] but FPT in parameter $h^{r}(\mathcal {T})$ [32]. That is, the question “Is $h^{r}(\mathcal {T}) \le k$?”, for some positive integer parameter *k*, can be answered in time $O( f(k) \cdot \text {poly}(|x|) )$ where *f* is a computable function that only depends on *k* and |*x*| is the size of the input instance *x* to HN. It is well-known that $h^{r}(\mathcal {T}) = 0$ if and only if all the trees in $\mathcal {T}$ are isomorphic, a task which can easily be done in polynomial time [29].

- **Problem:** Unrooted Hybridization Number (UHN)
- **Input:** A set $\mathcal {T}$ of unrooted binary phylogenetic trees on the same set of taxa *X*.
- **Output:** An unrooted binary phylogenetic network *N* on *X* such that, for each $T \in \mathcal {T}$, *N* displays *T*.
- **Goal:** Minimize *r*(*N*).

We write $h^u(\mathcal {T})$ to denote the minimum value of *r*(*N*) thus obtained. It is natural to ask: do feasible solutions to UHN require, as in the rooted case, certificates verifying that the input trees are displayed by the network? This motivates our study of the following problem, which we will start with in Sect. 3.

- **Problem:** Unrooted Tree Containment (UTC)
- **Input:** An unrooted binary phylogenetic network *N* and an unrooted binary phylogenetic tree *T*, both on *X*.
- **Question:** Does *N* display *T*?

Finally, we will consider the variant in which we require a root location to be determined for each of the unrooted input trees. A rooted binary phylogenetic tree $T'$ on *X* is a *rooting* of an unrooted binary phylogenetic tree *T* if $T'$ can be obtained by subdividing an edge of *T* with a new node *u* and directing all edges away from *u*. We say that *T* is the *unrooting* of $T'$, denoted $U(T')$.

- **Problem:** Root Uncertain Hybridization Number (RUHN)
- **Input:** A set $\mathcal {T}$ of unrooted binary phylogenetic trees on the same set of taxa *X*.
- **Output:** A root location (i.e. an edge) of each tree in $T \in \mathcal {T}$ (which induces a set of rooted binary phylogenetic trees $\mathcal {T'}$ on *X*) and a rooted binary phylogenetic network *N* on *X* such that, for each $T' \in \mathcal {T'}$, *N* displays $T'$.
- **Goal:** Minimize *r*(*N*).

The minimum value of *r*(*N*) obtained is denoted $h^{ru}(\mathcal {T})$ and is called the *root-uncertain hybridization number* of $\mathcal {T}$. Note that if $\mathcal {T}$ is a set of rooted binary phylogenetic trees and $\mathcal {T^{*}}$ is the set of unrooted counterparts of $\mathcal {T}$—that is, $\mathcal {T^{*}} = \{ U(T) | T \in \mathcal {T}\}$—then $h^{ru}(\mathcal {T^{*}})$ can differ significantly from $h^{r}(\mathcal {T})$. For example, if $\mathcal {T}$ consists of two rooted caterpillars on the same set of *n* taxa, but with opposite orientation, then $h^{r}(\mathcal {T}) = n-2$ whilst $h^{ru}(\mathcal {T^{*}}) = 0.$ More generally, given a set $\mathcal {T}$ of binary rooted trees and the set $\mathcal {T^*}$ of their corresponding unrooted versions, we have:

1 $$\begin{aligned} h^u(\mathcal {T^*}) \le h^{ru}(\mathcal {T^*}) \le h^{r}(\mathcal {T}). \end{aligned}$$

It is possible to say more about this inequality chain. Let $\mathcal {T^*}$ be the set of the two trees $T_1$ and $T_2$ shown in Fig. 1. It is easy to see that $h^{u}(\mathcal {T^*})=1$: we simply arrange the taxa in a circle with circular ordering *a*, *b*, *c*, *d*, *e*, *f* (see $N_u$ in Fig. 1). However, as can be verified by case analysis (or using the “re-root by hybridization number” functionality in Dendroscope [19]), $h^{ru}(\mathcal {T^*})=2$. Moreover, let $\mathcal {T}$ be the set of the two rooted trees obtained by rooting the first tree on the unique edge incident to *a*, and the second tree on the unique edge incident to *e*. It can be verified that $h^{r}(\mathcal {T})=3$. Hence, $\mathcal {T}$ is an example when both inequalities in (1) are simultaneously strict.

## The Tree Containment Problem on Unrooted Networks and Trees

Given a rooted binary phylogenetic network $N = (V,E)$ on *X* and a rooted binary phylogenetic tree *T* also on *X* it is trivial to determine in time $O( 2^k \cdot \text {poly}(n) )$ whether *N* displays *T*, where $k = r(N) = |E|- (|V|-1)$ and $n=|V|$. This is because, for each of the *k* reticulation nodes, we can simply guess which of its two incoming edges are on the image of *T*. Here we consider the natural unrooted analogue of the problem where both *N* and *T* are unrooted.

We show that the question whether *N* displays *T* is NP-hard, but FPT when parameterized by $k=r(N)=|E|-(|V|-1)$. Note that, unlike for the rooted case, an FPT result here is not trivial, since the notion “reticulation node” no longer has any meaning.

### The Hardness of Unrooted Tree Containment (UTC)

#### Theorem 1

UTC is NP-hard.

#### Proof

We reduce from the problem Node Disjoint Paths On Undirected Graphs (NDP). The reduction is similar in spirit to the reduction given in [20], where the hardness of tree containment on *rooted* networks was proven by reducing from NDP on *directed* graphs. However, our reduction has to deal with a number of subtleties specific to the case of unrooted trees and networks.

NDP is defined as follows. We are given an undirected graph $G=(V,E)$ and a multiset of unordered pairs of nodes $W=\{ \{s_1, t_1\}, \ldots , \{s_k, t_k\} \}$, where for each *i*, $s_i \ne t_i$. Note that we do not assume a distinction between the *s* nodes and the *t* nodes (we refer to them together as *terminals*), and the same pair can appear several times. The question is: do there exist *k* paths $P_i$ ($1 \le i \le k$) such that $P_i$ connects $s_i$ to $t_i$, and such that the $P_i$ are mutually node-disjoint?

The literature is somewhat ambiguous about whether endpoints of the paths are allowed to intersect, and of course this is a necessary condition if we are to allow some terminal to appear in more than one pair in *W*. We posit as few restrictions as possible on the input–specifically, we allow each terminal to be in multiple pairs—and then show that this can be reduced to a more restricted instance. We do however make use of the fact that NDP remains hard on cubic graphs^1^, and assume henceforth that *G* is cubic.

We start by first reducing the cubic NDP instance (*G*, *W*) to a new instance $(G',W')$ where $G'$ has maximum degree 3 and no nodes of degree 2, each terminal appearing within $W'$ is in exactly one pair, and a node of $G'$ is a terminal if and only if it has degree 1. As usual, the idea is that (*G*, *W*) is a YES instance for NDP if and only $(G',W')$ is. The transformation to $(G',W')$ is straightforward. Observe firstly that in the (*G*, *W*) instance each terminal can appear in at most 3 pairs (otherwise it is trivially a NO instance). Depending on whether a terminal is in 1, 2, or 3 pairs we use a different transformation.

**Fig. 2:**
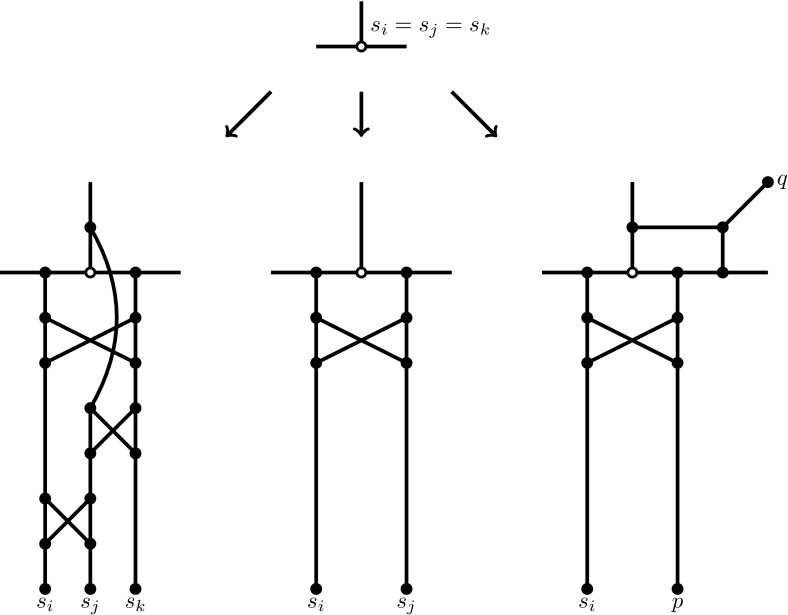
Gadgets for obtaining a transformed NDP instance $(G',W')$ where $G'$ has maximum degree 3, no nodes of degree 2, a node has degree 1 if and only if it is a terminal, and each terminal appears in exactly one pair

1. *A terminal is in 3 pairs in* *W* $\{s_i, t_i\}, \{s_j, t_j\}, \{s_k, t_k\}$ *where* $s_i = s_j= s_k$. We split the terminal into 3 distinct nodes; see Fig. 2 (left).
2. *A terminal is in 2 pairs in* *W* $\{s_i, t_i\}, \{s_j, t_j\}$ *where* $s_i = s_j$. We split the terminal into 2 distinct nodes; see Fig. 2 (middle).
3. *A terminal is in 1 pair in* *W* $\{s_i, t_i\}$. Here we do not split the terminal but we do introduce a new terminal pair $\{p,q\}$; see Fig. 2 (right). The introduction of $\{p,q\}$ concerns the fact that, prior to the transformation, at most one of the node disjoint paths can intersect with node $s_i$. The presence of $\{p,q\}$ ensures that, after transformation, at most one path can intersect with the image of this node. (A simpler transformation is not obviously possible, due to the degree restrictions on $G'$).

**Fig. 3:**
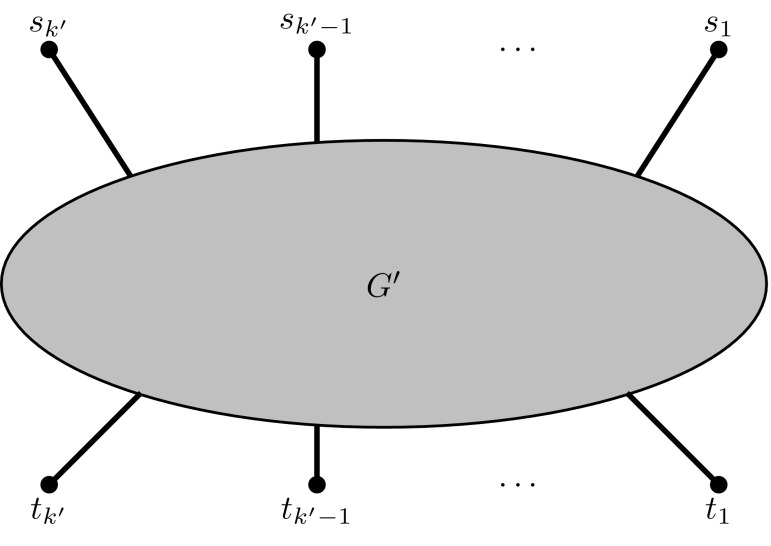
Schematic representation of the transformed NDP instance $(G',W')$

The transformations are applied as often as necessary to obtain the instance $(G',W')$. Let $W'=\{\{s_1, t_1\}, \ldots , \{s_{k'}, t_{k'}\}\}$. Due to the fact that each terminal now appears in exactly one pair, we can schematically view the $(G',W')$ instance as shown in Fig. 3.

**Fig. 4:**
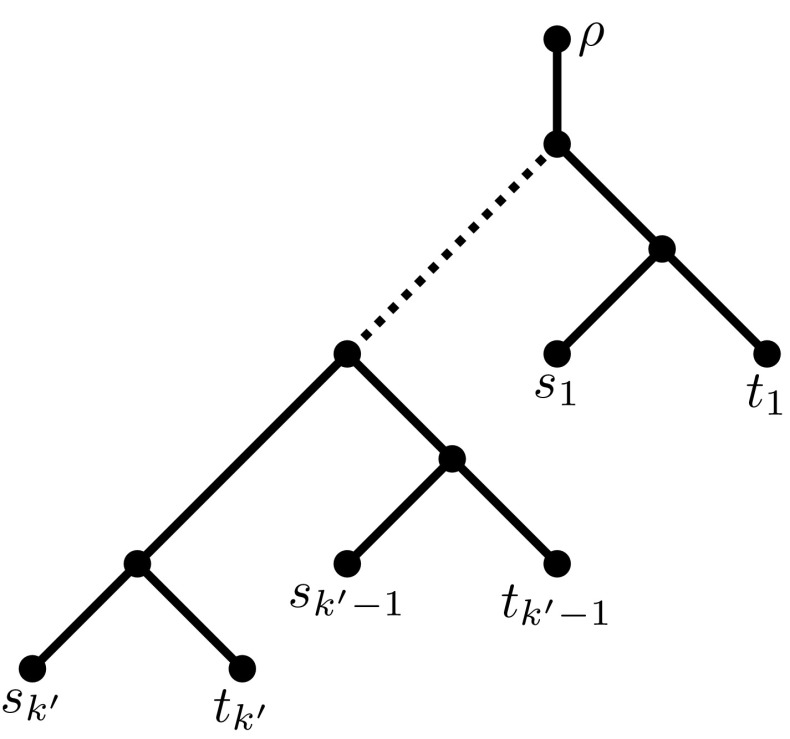
The tree *T* used in the reduction of NDP to UTC

**Fig. 5:**
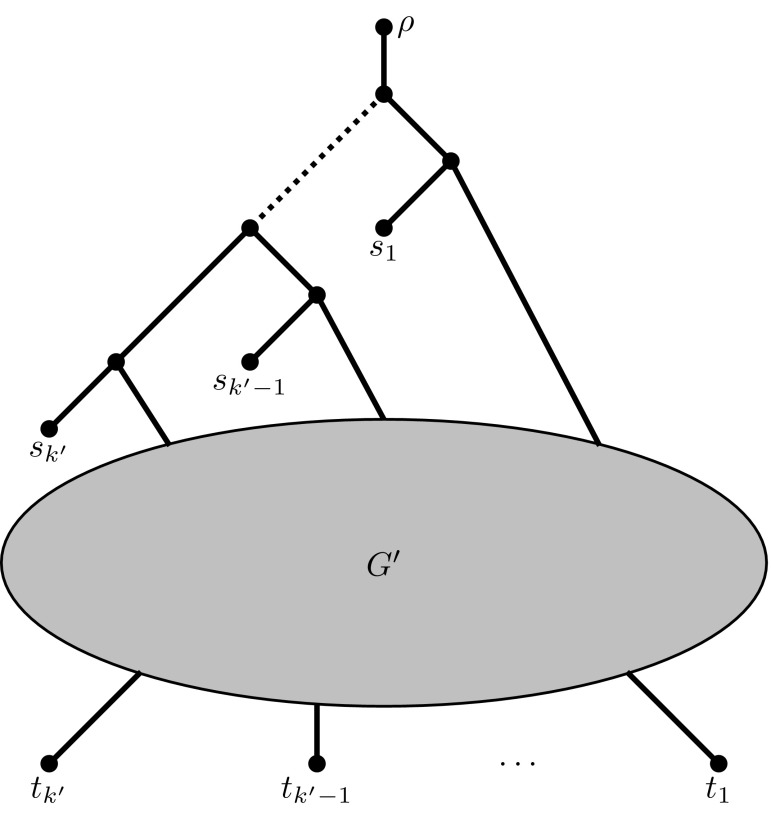
The network *N* used in the reduction of NDP to UTC

Now, we reduce $(G',W')$ to UTC. Let *T* be the unrooted binary phylogenetic tree on $2k'+1$ taxa $X = \{\rho , s_1, t_1, \ldots , s_{k'}, t_{k'} \}$ shown in Fig. 4. The unrooted binary phylogenetic network *N*, also on *X*, is constructed from $(G',W')$ as shown in Fig. 5. It can easily be verified that *N* displays *T* if and only if $(G',W')$ is a YES instance to NDP. $\square$

### Unrooted Tree Containment (UTC) Parameterized by Reticulation Number

Recall that the input to UTC is an unrooted binary phylogenetic network $N = (V,E)$ and an unrooted binary phylogenetic tree *T*, both on *X*. In this section we use $n := |V|$ to denote the size of the input to UTC, which is justified by noticing that $|X| \le |V|$ and $|V| - 1 \le |E| \le (3/2)|V|$ and that |*V*| can be arbitrarily larger than |*X*|.

We prove that UTC is fixed parameter tractable (FPT) in parameter *r*(*N*). First, we give a linear kernel: we show how to transform in $\text {poly}(n)$ time the instance (*N*, *T*) of ${\textsc {UTC}}$ into a new instance $(N'', T'')$ on $X''$ such that $r(N'') \le r(N)$, the size of the instance $(N'',T'')$ is at most a linear function of $r(N'')$, and $N''$ displays $T''$ if and only if *N* displays *T*. Second, we describe a simple bounded-search branching algorithm to answer UTC in time $O(4^{r(N)} n^2)$, and combining these two results establishes the FPT result. (Note that the second result alone is actually sufficient to establish the FPT result, and could be used without first applying the kernelization procedure, but the kernelization is of independent interest and can contribute to further speed-up in practice). It is important to mention here the recent result [16] which provides an $O(2^{0.694 \cdot r(N)}n)$-time algorithm for the *rooted* case of the TC problem. However, as discussed in the preliminaries, an unrooted network maps to potentially exponentially many rooted networks, so UTC cannot be efficiently reduced to the rooted case by simply guessing the root location within the unrooted network.

We start with some necessary definitions, which we give in a form somewhat more general than required in this section, so that we can use them in later sections.

Let $\mathcal {N}$ be a collection of binary, unrooted, phylogenetic networks (all on the same taxon set *X*) and $N_i \in \mathcal {N}$. Let $X' \subset X$. A subtree *T* is called a *pendant* subtree of $N_i$ if there exists an edge *e* the deletion of which detaches *T* from $N_i$. By $N_i|X'$ we mean the tree which is obtained from $N_i$ by taking the *minimum spanning tree* on $X'$ and then suppressing any resulting node of degree 2. Also, let $e_i$ be the edge of network $N_i \in \mathcal {N}$ the deletion of which detaches the pendant subtree *T* from $N_i$ and let $v_i \in e_i$ be the endpoint of $e_i$ “closest” to the taxon set $X'$, where $X' \subset X$ is the set of taxa induced by the subtree *T*. Let’s say that we root each $N_i|X'$ at $v_i$, thus inducing a rooted binary phylogenetic tree $(N_i|X')^{\rho }$ on $X'$.

A subtree *T*, inducing a subset of taxa $X' \subset X$, is called *common pendant subtree of* $\mathcal {N}$ if the following two conditions hold:

1. *T* is a pendant subtree on each $N_i \in \mathcal {N}$ and $N_i | X' = N_j|X'$ for each pair of two distinct networks $N_i, N_j \in \mathcal {N}$.
2. We require that, for each distinct pair of networks $N_i, N_j \in \mathcal {N}$, the following to be true: $(N_i|X')^{\rho } = (N_j|X')^{\rho }$.

The second condition formalizes the idea that the point of contact (root location) of the tree should explicitly be taken into account when determining whether a pendant subtree is common. (This is consistent with the definition of common pendant subtree elsewhere in the literature).

The above definition is the basis of the following polynomial-time reduction rule which we will use extensively.

- *Common Pendant Subtree (CPS) reduction* Find a maximal common pendant subtree in $\mathcal {N}$. Let *T* be such a common subtree with at least two taxa and let $X_T$ be its set of taxa. Clip *T* from each $N_i \in \mathcal {T}$. Attach a single label $x \notin X$ in place of *T* on each $N_i$. Set $X := (X {\setminus } X_T) \cup \{x\}$.

Note that, if all the networks in $\mathcal {N}$ are copies of the same, identical unrooted binary tree on *X*, we adopt the convention that iterated application of the CPS reduction reduces all the trees to a single taxon set $X = \{x\}$.

Next, let *N* be an unrooted binary network on *X*. For each taxon $x_i \in X$, let $p_i$ be the unique parent of $x_i$ in *N*. Let $C = (x_1, x_2, \ldots , x_t)$ be an ordered sequence of taxa and let $P = (p_1, p_2, \ldots , p_t)$ be the ordered sequence corresponding to their parents. We allow $p_1 = p_2$ or $p_{t-1} = p_t$. If *P* is a *path* in *N* then *C* is called a *chain* of length *t*. A chain *C* is a *common chain* of $\mathcal {N}$ if *C* is a chain in each $N_i \in \mathcal {N}$. This brings us to our second polynomial-time reduction rule.

- *Common* *d*-*Chain* (*d*-*CC*) *reduction* Find a maximal common chain $C = (x_1, \ldots , x_t)$ of $\mathcal {N}$ where $t > d$. Delete from each $N_i \in \mathcal {N}$ all leaf labels $x_{d+1}, \ldots , x_t$, suppress any resulting node of degree 2 and delete any resulting unlabelled leaves of degree 1.

We now add a third reduction rule which helps to further reduce the size of the kernel and (more importantly) the value of the parameter (i.e., the reticulation number). We assume that none of the previous reduction rules are applicable. We make a similar assumption in the proof below that this reduction rule is safe.

- *Network Chain (NC) reduction* Let (*N*, *T*) be an instance to the UTC problem where *N* is an unrooted binary phylogenetic network and *T* is an unrooted binary phylogenetic tree, both on *X*. If the network *N* contains a chain $(x_1,\ldots ,x_t)$ with $t\ge 3$ then proceed as follows. Let $e_{i,i+1}$ be the edge connecting the parents of $x_i$ and $x_{i+1}$. Let $e_1$ be the edge incident to the parent of $x_1$ that is not $e_{12}$ and not incident to $x_1$. Let $e_t$ be the edge incident to the parent of $x_t$ that is not $e_{t-1,t}$ and not incident to $x_t$. (Note that all these edges exist, because the network does not contain any pendant subtrees, and thus no pendant chains.)
  1. If $t\ge 7$ then return a trivial NO instance to the UTC problem.
  2. If $t=6$ then delete $e_{34}$.
  3. If $t=5$, do the following. If the tree contains a chain $(x_1,x_2,x_3)$, delete $e_{34}$. Otherwise, delete $e_{23}$.
  4. If $t=4$, do the following. If the tree contains a chain $(x_1,x_2,x_3)$, delete $e_{34}$. If it contains a chain $(x_2,x_3,x_4)$, delete $e_{12}$. Otherwise, delete $e_{23}$.
  5. If $t=3$ and the tree has a pendant subtree on $\{x_1,x_2,x_3\}$, do the following. If $x_1$ and $x_2$ have a common parent in the tree, delete $e_1$. Otherwise, delete $e_3$.
  6. Otherwise, if $t=3$ and the tree has a pendant subtree on $\{x_1,x_2\}$, delete $e_{23}$.
  7. Otherwise, if $t=3$ and the tree has a pendant subtree on $\{x_2,x_3\}$, delete $e_{12}$.
  8. Otherwise, if $t=3$ and the tree has a chain $(x_1,x_2,x_3)$, delete $x_3$.
  In all cases, we suppress any resulting degree-2 nodes.

If, during the kernelization procedure, we ever discover that the answer to UTC is definitely NO (respectively, YES) then we simply output a trivial NO (resp. YES) instance as $(N'',T'')$ e.g. letting $N''$ and $T''$ be two topologically distinct (resp. identical) unrooted phylogenetic trees on 4 taxa and 5 edges. We shall henceforth use this implicitly; this is where the “4” and “5” terms come from in the statement of Lemma 3. Note that if $|X| \le 3$, the answer is trivially YES, so we henceforth assume $|X| \ge 4$.

We begin with some trivial pre-processing. If *N* contains a cut-edge *e* such that one of the two connected components obtained by deleting *e* contains no taxa, we delete *e* and this component from *N* and suppress the degree 2 node created by deletion of *e*. (This is safe, i.e. does not alter the YES/NO status of the answer to UTC because the image of *T* in *N* can never enter such a component). We repeat this step until it no longer holds. Let $N'$ be the resulting network. If $N'$ and *T* are both trees, and are topologically distinct (resp. identical) the answer is definitely NO (resp. YES). Hence, we assume that $N'$ is not a tree.

In the next two lemmas we will show that (1) the **Common Pendant Subtree (CPS) reduction** and the **Common 3-Chain (3-CC) reduction** rules are safe and (2) the **Network Chain (NC) reduction** rule is also safe.

#### Lemma 1

The application of (CPS) and (3-CC) rules is safe.

#### Proof

We apply the (CPS) reduction rule to $(N',T)$ until it can no longer be applied. It is easy to see that applying this reduction is safe. This is because the image of the common pendant subtree, and the common pendant subtree itself, are necessarily identical in $N'$. Gently abusing notation, let $N'$ be the resulting network and $T'$ the resulting tree. Observe that at this stage $N'$ potentially still contains pendant subtrees (with 2 or more taxa). This occurs if the pendant subtree has no common counterpart in $T'$. However, if this happens the answer is definitely NO. Therefore, we can henceforth assume that $N'$ contains no pendant subtrees (with 2 or more taxa).

The next step is to apply the (3-CC) reduction rule repeatedly to $(N',T')$ until it can no longer be applied. This has the effect of clipping all common chains on 4 or more taxa to length 3. (The fact that we can clip common chains to constant length is the reason we obtain a linear kernel). Let $(N'', T'')$ be the instance obtained after a single application of the (3-CC) reduction rule. To establish correctness it is sufficient to show that $(N'',T'')$ is a YES instance if and only if $(N',T')$ is a YES instance.

It is easy to see that if $(N', T')$ is a YES instance then $(N'', T'')$ is a YES instance. This is because, if $N'$ contains an image of $T'$, then an image of $T''$ (in $N''$) can be obtained from the image of $T'$ simply by disregarding the surplus taxa deleted from the chain.

**Fig. 6:**
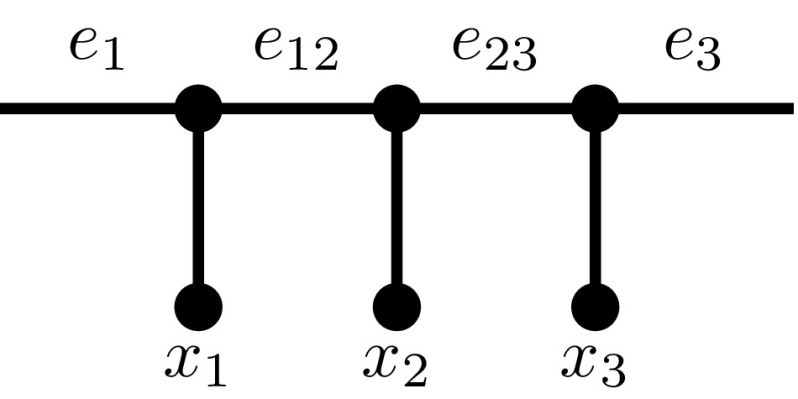
The chain $C''$ in $N''$

The other direction is somewhat more subtle. Observe that, prior to the chain reduction, the common chain $C' = (x_1, x_2, \ldots , x_t)$, $t \ge 4$, was not pendant in $N'$ (because $N'$ contained no pendant subtrees). Hence, the clipped chain $C'' = (x_1, x_2, x_3)$ is not pendant in $N''$. Let $e_1, e_{12}, e_{23}, e_{3}$ be the 4 interior edges of $N''$ shown in Fig. 6.

Now, suppose $N''$ displays $T''$; we will prove that $N'$ displays $T'$. Fix some image of $T''$ inside $N''$. We distinguish two main cases. Note that $C'$ is pendant in $T'$ if and only if $C''$ is pendant in $T''$.

*Case 1* $C''$ *is* not *pendant in* $T''$. Both $e_1$ and $e_3$ must be on the image of $T''$ in $N''$, because otherwise the image of the chain $C''$ is pendant, a contradiction. If both $e_{12}$ and $e_{23}$ are also on the image, then the chain $C''$ and its image in $N''$ are identical. In particular, there is no ambiguity about the orientation of the chain, so reintroducing the clipped taxa $(x_4, \ldots , x_t)$ into the image of $T''$ (next to $x_3$) yields an image of $T'$ in $N'$. The only remaining subcase is that, in addition to both $e_1$ and $e_3$, exactly one of $\{e_{12}, e_{23}\}$ is on the image. Without loss of generality let this be $e_{12}$. However, this is not possible, because it would mean that $\{x_1, x_2\}$ are pendant in the image of $C''$, and this cannot be an image of $T''$ because $\{x_1, x_2\}$ are not pendant in $T''$.

*Case 2* $C''$ is *pendant in* $T''$. There are two subcases to consider.

- In the first subcase, $x_1$ and $x_2$ share a parent in $T''$. (That is, the chain is oriented *towards* the rest of the tree). In such a situation both $e_{12}$ and $e_{3}$ must be on the image of $T''$. (If this was not the case, $\{x_2, x_3\}$ would be pendant in the image of $C''$, but this is not possible because they are not pendant in $T''$.) Now, if $e_{23}$ is on the image (irrespective of whether $e_1$ is on the image) then, as in the earlier case, reintroducing the clipped taxa $(x_4, \ldots , x_t)$ into the image of $T''$ (next to $x_3$) yields an image of $T'$ in $N'$. The main subtlety is if $e_{23}$ is not on the image, and (necessarily) $e_1$ is. This occurs if the image of $C''$ exits via $e_1$, follows some simple path *P* through another part of the network, and re-enters at $e_3$. However, note that, within the image, the path *P* contains exactly one node of degree 3—which is the image of the parent of $x_3$—and for the rest only degree 2 nodes. This means that we can manipulate the image of $T''$ as follows: put $e_{23}$ in the image, remove $e_1$ from the image, and then tidy up the image in the usual sense (i.e. repeatedly deleting unlabelled nodes of degree 1). This is a new, valid image of $T''$, and puts us back in the situation when $e_{23}$ *is* on the image, so we are done.
- In the second subcase, $x_2$ and $x_3$ share a parent in $T''$. (That is, the chain is oriented *away* from the rest of the tree). Observe that $e_1$ and $e_{23}$ must be on the image, because otherwise $\{x_1, x_2\}$ is pendant in the chain image but not in $T''$. If $e_{12}$ is in the image (irrespective of whether $e_3$ is in the image), re-introducing the clipped taxa $(x_4, \ldots , x_t)$ to the right of $x_3$ yields an image of $T'$ in $N'$. Again, there is one subtle situation, and that is when $e_{12}$ is not on the image, but $e_3$ is. Just as before this occurs if the image of $C''$ exits via $e_1$, follows some simple path *P* through another part of the network, and re-enters at $e_3$. The unique node on *P* of degree 3 is the image of the parent of $x_1$ (and all other nodes on *P* are degree 2). Hence, if we put $e_{12}$ into the image, remove $e_3$ from the image, and tidy the image up, we obtain a new valid image of $T''$ and we are back in the case when $e_{12}$ is in the image.

Thus, we have established that if $N''$ displays $T''$, then $N'$ displays $T'$. Hence, an application of the 3-CC chain reduction is always safe. $\square$

We now show that the NC reduction rule is safe.

#### Lemma 2

Assume that neither of the (CPS), (3-CC) reduction rules can be applied. Then the (NC) reduction rule is always safe to apply.

#### Proof

Suppose that the network displays the tree. Then the chain $(x_1,\ldots ,x_t)$ of the network is either also a chain of the tree, or there exists some $1\le i\le t-1$ such that the tree has pendant chains on $\{x_1,\ldots ,x_i\}$ and on $\{x_{i+1},\ldots ,x_t\}$. We now discuss each case of the network chain reduction separately.

1. In this case it follows that there is a common chain of length at least four, which is not possible since we assumed that the (3-CC) reduction rule is not applicable.
2. This is only possible if $(x_1,x_2,x_3)$ and $(x_4,x_5,x_6)$ are pendant chains of the tree. Hence, $e_{34}$ is not used by any image of the tree in the network and can be deleted.
3. If the tree contains a chain $(x_1,x_2,x_3)$, then it must be pendant. Hence, $e_{34}$ can be deleted. Otherwise, $(x_3,x_4,x_5)$ must be a pendant chain of the tree and $e_{23}$ can be deleted.
4. Similar to the previous case. If neither $(x_1,x_2,x_3)$ nor $(x_2,x_3,x_4)$ is a pendant chain of the tree, then $(x_1,x_2)$ and $(x_3,x_4)$ must both be pendant chains of the tree, in which case $e_{23}$ can be deleted.
5. Similar to the previous cases.
6. In this case, $(x_1,x_2,x_3)$ is a chain of the tree that is not pendant (since otherwise we would be in one of the previous cases). The image of the tree in the network must then use all of $e_1,e_{12},e_{23},e_3$. Now we delete $x_3$ and suppress the resulting degree-2 node. Hence the reduced network has a chain $(x_1,x_2)$ with edges $e_1,e_{12},e_2$ defined as in the network chain reduction rule. To see that this reduction is safe, assume that the reduced tree is displayed by the reduced network. Then the embedding of the tree in the network has to use $e_1$ and $e_2$. It does not necessarily use $e_{12}$ but if it does not it is easy to adapt the image such that it does use $e_{12}$. Hence, the chain $(x_1,x_2)$ can be replaced by $(x_1,x_2,x_3)$ and it follows that the original tree is displayed by the original network.

$$\square $$

#### Lemma 3

There exists a kernelization for UTC producing an instance $(N'',T'')$ with at most $\max ( 6k, 4 )$ taxa and $\max ( 15k, 5)$ edges, where $k = r(N'') \le r(N)$.

#### Proof

Let $(N'',T'')$ be an instance obtained by applying the (CPS), (3-CC) and (NC) reduction rules exhaustively until none applies. Clearly, the process by which $(N'',T'')$ is obtained from the original (*N*, *T*) can be completed in polynomial time, since all pre-processing steps delete at least one node or edge from the network. It is easy to verify that, by construction, $r(N'') \le r(N)$. Hence, to complete the kernelization it remains only to show that the size of the instance $(N'',T'')$ is at most a linear function of $r(N'')$, where for brevity we let $k = r(N'')$. To see this, recall firstly that $N''$ has no pendant subtrees. Let $N'' = (V'',E'')$. Suppose we delete all taxa in $N''$ and then repeatedly suppress nodes of degree 2, and delete nodes of degree 1, until neither of these operations can be applied anymore. For $k\ge 2$, this creates a 3-regular graph $N^{*}$ with nodes $V^{*}$ and edges $E^{*}$, that potentially contains multi-edges and loops. Notice that in each deletion of a leaf and each suppression of a node with degree 2, we diminish both the number of nodes and the number of edges by 1. Since we started out with $|E''|=k+|V''|-1$ we still have $|E^{*}|=k+|V^{*}|-1$. Moreover, because of 3-regularity, $|E^{*}|=3|V^{*}|/2$. Combining yields $|V^{*}|=2k-2$ and therefore $|E^{*}|=3(k-1)$. (For $k=1$, $N^{*}$ contains no nodes and is strictly speaking not a graph: in this case we define $N^{*}$ to be a single node with a loop). Observe that $N''$ can be obtained from $N^{*}$ by replacing each edge with a chain of taxa: this operation is sufficient because $N''$ had no pendant subtrees. Moreover, each such chain contains at most two leaves since otherwise the network chain reduction rule would be applicable. This means that $|X''|$ is at most $2 \cdot \max (1, 3(k-1))$, and the number of edges in $N''$ is at most $5 \cdot \max (1, 3(k-1))$.

**Fig. 7:**
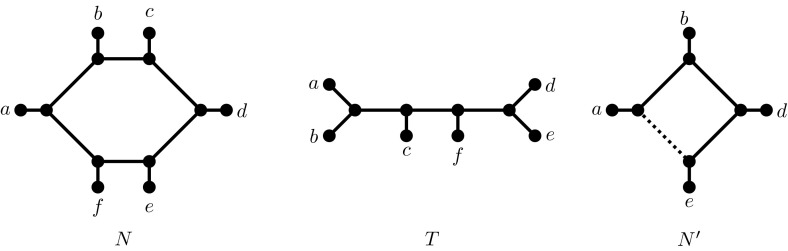
Example showing that it is not safe to reduce chains to length 2. The shown network *N* does not display the given tree *T*. However, if the chains (*a*, *b*, *c*) and (*d*, *e*, *f*) are reduced to length 2, then the reduced network $N'$ does display the reduced tree $T'$ (by deleting the *dotted edge*)

We observe that simply reducing common chains to length 2, i.e. applying the (2-CC) reduction rule, is *not* safe, as the following example shows. Suppose *N* consists of a single cycle with taxa *a*, *b*, *c*, *d*, *e*, *f* in that (circular) order. Let *T* be a caterpillar tree with taxa *a*, *b*, *c*, *f*, *e*, *d* in that order. *N* does not display *T*. However, if the common chain (*a*, *b*, *c*) is clipped to (*a*, *b*)—or to (*b*, *c*)—the resulting network $N''$ *does* display $T''$. A symmetrical argument also holds for the common chain (*f*, *e*, *d*) (Fig. 7).

The proof of the FPT result follows by applying a simple bounded-search branching algorithm to the kernelized instance. Note that, as mentioned earlier, this algorithm can be applied independently of the kernelization.

#### Theorem 2

Let (*N*, *T*) be an input to UTC, where $N=(V,E)$. There exists an $O(4^kn^2)$-time algorithm for UTC, where $k= r(N)$ and $n = |V|$.

#### Proof

If the network is a tree then the problem can be solved easily in polynomial time by deciding whether or not the network is isomorphic to the input tree. Otherwise, we proceed as follows.

Consider any two taxa *x*, *y* that have a common neighbour in the tree *T*. If *x* and *y* also have a common neighbour in *N*, then we can delete *y* from both *T* and *N* and suppress the resulting degree-2 nodes (see the (CPS) reduction above).

Otherwise, let $n_x$ and $n_y$ be the neighbours of, respectively, *x* and *y* in the network *N*. Let $e_1,e_2$ be the two edges that are incident to $n_x$ but not to *x* and let $e_3,e_4$ be the two edges that are incident to $n_y$ but not to *y*. If *N* displays *T*, then the embedding of *T* in *N* can contain at most three of these four edges $e_1,\ldots ,e_4$ (since there is exactly one edge leaving the path between *x* and *y* in the embedding). Hence, we create four subproblems $P_1,\ldots ,P_4$. In subproblem $P_i$, we delete edge $e_i$ and suppress resulting degree-2 nodes. The parameter (reticulation number) in each subproblem is $k-1$. Hence, the running time is $O(4^kn^2)$.

## Unrooted Hybridization Number (UHN) on Two Trees

In this section we study the unrooted hybridization number problem in case the input consists of two trees $T_1, T_2$ and we show equivalence to a well-known problem that has been studied before in the literature, namely the *tree bisection and reconnect* problem.

Let *T* be an unrooted, binary tree on *X*. A *tree bisection and reconnect* (TBR) move is defined as follows: (1) we delete an edge of *T* to obtain a forest consisting of two subtrees $T'$ and $T''$. (2) Then we select two edges $e_1 \in T', e_2 \in T''$, subdivide these two edges with two new nodes $v_1$ and $v_2$, add an edge from $v_1$ to $v_2$, and finally suppress all nodes of degree 2. In case either $T'$ or $T''$ are single leaves, then the new edge connecting $T'$ and $T''$ is incident to that node. Let $T_1, T_2$ be two binary unrooted phylogenetic trees on the same set of leaf-labels. The TBR-distance from $T_1$ to $T_2$, denoted $d_{TBR}(T_1, T_2)$, is simply the *minimum* number of TBR moves required to transform $T_1$ into $T_2$.

It is well known that computation of TBR-distance is essentially equivalent to the Maximum Agreement Forest (MAF) problem, which we now define. Given an unrooted, binary tree on *X* and $X' \subset X$ we let $T(X')$ denote the minimal subtree that connects all the elements in $X'$. An *agreement forest* of two unrooted binary trees $T_1, T_2$ on *X* is a partition of *X* into non-empty blocks $\{X_1, \ldots , X_k\}$ such that (1) for each $i \ne j$, $T_1(X_i)$ and $T_1(X_j)$ are node-disjoint and $T_2(X_i)$ and $T_2(X_j)$ are node-disjoint, (2) for each *i*, $T_1|X_i = T_2|X_i$. A *maximum agreement forest* is an agreement forest with a minimum number of components, and this minimum is denoted $d_{MAF}(T_1,T_2)$. In 2001 it was proven by Allen and Steel that $d_{MAF}(T_1, T_2) = d_{TBR}(T_1, T_2) + 1$ [1].

### Theorem 3

Let $T_1, T_2$ be two unrooted binary phylogenetic trees on the same set of taxa *X*. Then $d_{TBR}(T_1, T_2) = h^u(T_1,T_2)$.

### Proof

We first show $h^u(T_1,T_2) \le d_{TBR}(T_1,T_2)$. Let $d_{TBR}(T_1,T_2)=k$. Observe that if $k=0$ then $T_1 = T_2$, because $d_{TBR}$ is a metric, and if $T_1 = T_2$ then $h^{u}(T_1, T_2)=0$, so the claim holds. Hence, assume $k \ge 1$.

By the earlier discussed equivalence, $T_1$ and $T_2$ have an agreement forest with $k+1$ components $F = \{F_0, \ldots , F_{k}\}$. Our basic strategy is to start with a network that trivially displays $T_1$ (specifically, $T_1$ itself) and then to “wire together” the components of *F* such that an image of $T_2$ is progressively grown. Each such wiring step involves subdividing two edges and introducing a new edge between the two subdivision nodes. This increases the number of nodes in the network by 2 and the number of edges by 3, so it increases the reticulation number by 1. We will do this *k* times, yielding the desired result.

Observe that for least one of the components, $F_{p}$ say, $T_2(F_p)$ will be pendant in $T_2$. Let $F' = F {\setminus } \{F_p\}$, $X' = X {\setminus } F_{p}$, $T'_1 = T_1|X'$ and $T'_2 = T_2|X'$. Let $\{u,v\}$ be the edge that, when deleted, detaches $T_2(F_{p})$ from the rest of the tree. Assume without loss of generality that *u* lies on $T_2(F_{p})$ and *v* lies on $T_2( X' )$. The nodes *u* and *v* thus lie on unique edges of $T_2 | F_p$ and $T_2 | X'$ (or taxa if $F_p$ and/or $X'$ are singleton sets); these can be viewed as the wiring points where $F_p$ wants to connect to the rest of the tree. Next, observe that $F'$ is an agreement forest for $T'_1$ and $T'_2$, so it too has a pendant component, and the process can thus be iterated. In this way we can impose an elimination ordering on the components of *F*. For the sake of brevity assume that the components $F_0, F_1, \ldots , F_k$ are already ordered in this way.

**Fig. 8:**
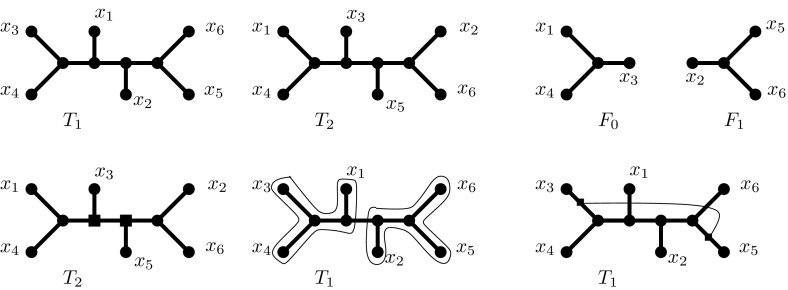
An illustration of the constructive proof of Theorem 3. We have the two trees $T_1, T_2$ shown in the *upper left* and *upper middle part* of the figure, respectively. In the *upper right part* of the figure we have an agreement forest consisting of two components $F_0,F_1$ that corresponds to the following bipartition of the leaf set: $F_0 = \{x_1,x_3,x_4\}, F_1 = \{x_2,x_5,x_6\}$. The main idea is, starting from $T_1$, to find the images of $T_2|F_0, T_2|F_1$ in $T_1$ (*lower middle part*). After we locate these images, we connect the images of the “interface” points of these two components in $T_2$ (shown in *big squares* in $T_2$ in the *lower left*, and in *small squares* in $T_1$ in the *lower right* part of the figure). This way we guarantee that the constructed network displays $T_2$ while we increase the reticulation number of $T_1$ by exactly 1. The second part of the proof simulates the opposite direction of the above construction

Now, set $N_k := T_1$. For each $F_i \in F$, fix the unique image of $F_i$ in $N_k$ (this allows us without ambiguity to refer to *the* image of $F_i$ in the intermediate networks we construct). For each $0 \le j \le k-1$, we construct $N_{j}$ from $N_{j+1}$ in the following way. Assume that by construction $N_{j+1}$ already contains an image of $T_2 | (\cup _{j' > j} F_{j'})$ and an image of $T_2|F_j$, and that these images are disjoint. (Clearly this is true for $j=k-1$, by the definition of agreement forest). From the earlier argument we know the two wiring points at which $T_2 | F_j$ wishes to join with $T_2 | (\cup _{j' > j} F_{j'})$. If $|F_j| \ge 2$ the wiring point within $F_j$ will be an edge, otherwise it is a taxon, and an identical statement holds for $|\cup _{j' >j} F_{j'}|$. Assume for now that both wiring points are edges, $e_1$ and $e_2$ respectively. The images of these edges will, in general, be paths in $N_{j+1}$. We subdivide any edge on the image of $e_1$, and any edge on the image of $e_2$, and connect them by a new edge. If a wiring point is a taxon *x* the only difference is that we subdivide the unique edge entering *x* in $N_{j+1}$. At the end of this process, $N_0$ displays both $T_1$ and $T_2$. This completes the claim $h^{u}(T_1,T_2) \le d_{TBR}(T_1,T_2)$.

To prove $h^{u}(T_1,T_2) \ge d_{TBR}(T_1,T_2)$, let $k = h^u(T_1,T_2)$ and let *N* be an unrooted phylogenetic network with reticulation number *k* that displays both $T_1$ and $T_2$. Fix an image $T'_1$ of $T_1$ inside *N*. If this image is not a spanning tree of *N*, greedily add edges to the image until it becomes one. (The edges added this way will correspond to unlabelled degree 1 nodes that are repeatedly deleted when tidying up the image to obtain $T_1$). Now, fix an image $T'_2$ of $T_2$ inside *N*. Let $F\subseteq E(N)$ be those edges of *N* that are *only* in $T'_2$. Deleting in $T_2$ the edges that correspond to *F* breaks $T_2$ up into a forest with at most $|F|+1$ components. In fact, by construction this will be an agreement forest. Hence, $d_{TBR}(T_1, T_2) \le |F|$. What remains is to show that $|F| \le h^{u}(T_1,T_2)$. Given that $T'_1$ was a spanning tree of *N*, and none of the edges on this image are in *F*, the graph $(V, E {\setminus } F)$ is connected, so $|E|-|F| \ge |V|-1$. Hence, $|F| \le |E|-|V|+1 = k$.

Note that the proof given above is constructive, in the following sense. Given an agreement forest *F* with $k+1$ components, one can easily construct in polynomial time an unrooted network *N* with reticulation number *k* that displays both the trees, and given an unrooted network *N* with reticulation number *k*, and images of $T_1$ and $T_2$ in *N*, one can easily construct in polynomial time an agreement forest *F* with $k+1$ components. An illustration of the main ideas involved to prove Theorem 3 can be found in Fig. 8.

### Corollary 1

UHN is NP-hard, in APX, and FPT in parameter $h^{u}(T_1, T_2)$.

### Proof

Immediate from Theorem 3 and the corresponding results for $d_{TBR}$. Hardness (and a linear-size kernel) were established in [1]. The best-known approximation result for $d_{TBR}$ is currently a polynomial-time 3-approximation [35, 36]. The best-known FPT result for $d_{TBR}$ is currently $O( 3^{k} \cdot \text {poly}(n))$ [8].

## Root-Uncertain Hybridization Number (RUHN)

In this section we turn our attention to the Root Uncertain Hybridization Number (RUHN) problem. We remind the reader that in this problem the input consists of a set of *unrooted* binary trees and we are asked to choose the root location of each tree, such that the hybridization number is minimized. In the first part of this section we show that RUHN is already NP-hard and APX-hard even when the input consists of two trees. On the other hand, in the next subsection we show that the problem is FPT in the hybridization number for any number of trees by providing a quadratic-sized kernel. We conclude the section by discussing how an exponential-time algorithm can be obtained for solving the kernel.

### Hardness

#### Lemma 4

Let $\mathcal {T} = \{ T_1, T_2 \}$ be an input to HN. We can transform in polynomial time $T_1$ and $T_2$ into two unrooted binary phylogenetic trees $T^{*}_1$, $T^{*}_2$ such that,

2 $$\begin{aligned} h^{ru}( T_1^{*}, T_2^{*} ) = h^{r}( T_1, T_2) + 1. \end{aligned}$$

#### Proof

Let *X* denote the taxa of $T_1$ and $T_2$ and let $n = |X|$. We will construct in polynomial time a pair of unrooted trees $T_1^{*}, T_2^{*}$ on $3|X|+2$ taxa such that (2) holds.

**Fig. 9:**
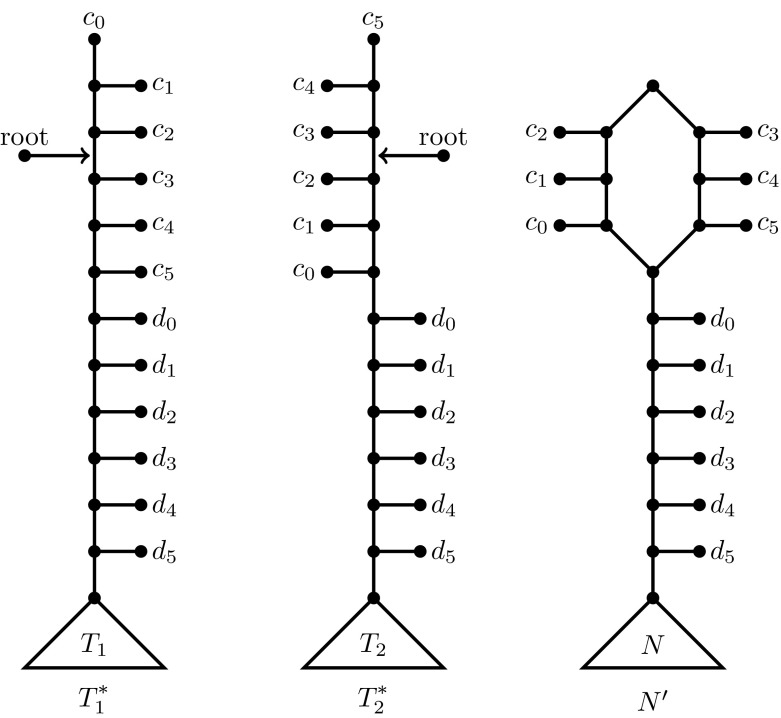
An example of the transformation used in Lemma 4 when $|X|=5$. *Left* and *centre* the two unrooted binary trees $T^{*}_1$ and $T^{*}_2$ that are used as input to RUHN. These are obtained from the original rooted binary trees $T_1$ and $T_2$ on *X* that are the input to the HN problem. If these trees are rooted at the specified points, then the rooted phylogenetic network $N'$ displays the two rootings, where *N* is an optimal solution to the original *HN* problem (although not shown explicitly here, in the top part of $N'$ all arcs are oriented downwards)

To construct $T^{*}_1$, we start by taking an unrooted caterpillar $(c_{0}, c_{1}, \ldots , c_n, d_0, d_1,$ $d_2, \ldots , d_{n})$ on $2n+2$ new taxa. Let $r_1$ be the root of $T_1$. To complete $T_1^{*}$ we ignore all the directions on the arcs of $T_1$, and concatenate the caterpillar to $T_1$ by subdividing the unique edge entering $d_n$ with a new node *u*, and connect *u* to $r_1$. The construction of $T^{*}_2$ is analogous, except that the *c*-part of the caterpillar is reversed: $(c_n, c_{n-1}, \ldots , c_0, d_0, d_1, d_2\ldots , d_n)$. See Fig. 9 (left and centre) for an example when $n=5$.

It is quite easy to show that $h^{ru}( T_1^{*}, T_2^{*} ) \le h^{r}( T_1, T_2) + 1$. Specifically, let *N* be any optimum solution to the original HN problem, i.e. $r(N) = h^{r}(T_1, T_2)$. If we root both $T^{*}_1$ and $T^{*}_2$ on the internal edge between $c_{2}$ and $c_3$, then the network $N'$ as shown in Fig. 9 (right) clearly displays these two rootings. Essentially, $N'$ has been obtained by adding a single “root cycle” above *N*, so $r(N') = r(N)+1$. More formally, in order of increasing distance from the root, the network $N'$ has taxa $c_2, c_1, c_0$ on one side of the root cycle, and $c_{3}, \ldots , c_{n-1}, c_{n}$ on the other.

The lower bound, $h^{ru}(T^{*}_1, T^{*}_2) \ge h^{r}(T_1, T_2) + 1$, requires slightly more effort to prove. We will use the following observation.

3 $$\begin{aligned} h^{ru}(T^{*}_1, T^{*}_2) \le h^{r}(T_1, T_2) + 1 \le (n-2) + 1 = n-1. \end{aligned}$$

The second inequality follows from the well-known fact that two rooted binary phylogenetic trees on $n \ge 2$ taxa can have hybridization number at most $n-2$ [2].

Notice that, if in a rooting of $T^{*}_1$, the whole *c*-part of the caterpillar appears in reverse order of the one in a rooting of $T^{*}_2$ then just this *c*-part of the caterpillars adds $n-1$ to the hybridization number of that rooting. The same holds for the *d*-parts of the caterpillars. In both cases, using the observation above, the lower bound is true. In particular, this implies that the lower bound holds if $T^{*}_1$ is rooted inside its $T_1$-part, since any rooting of $T^{*}_2$ will create either oppositely-oriented *c*- or *d*-parts of the caterpillars. The same holds for a rooting inside $T_2$. But clearly, if both $T^{*}_1$ and $T^{*}_2$ are rooted outside their *T*-parts, then these *T*-parts add $h^{r}(T_1, T_2)$ to the hybridization number of such a rooting. Since the caterpillars of $T^{*}_1$ and $T^{*}_2$ are non-isomorphic, any rooting within the *c*- or *d*-parts of caterpillars of the trees will additionally add at least 1 to the hybridization number. (Formally speaking this last argument is a consequence of the *cluster reduction* described in [3]).

#### Theorem 4

Root Uncertain Minimimum Hybridization is NP-hard and APX-hard for $|\mathcal {T}|=2$.

#### Proof

HN is already known to be NP-hard and APX-hard for $|\mathcal {T}|=2$. NP-hardness of RUHN is thus immediate from Lemma 4. We can also use this lemma to prove APX-hardness, which excludes the existence of a PTAS for RUHN, unless P=NP. The APX-hardness result might seem intuitively obvious, since the $+1$ term in (2) is of vanishing significance as $h^{r}(T_1, T_2)$ grows. However, there are quite some technicalities involved in the extraction of a solution for HN from a solution for RUHN. In particular, additional combinatorial insight is required. We give a (2, 1) L-reduction from HN to RUHN. In fact, this can be extended to an $(\alpha ,1)$ L-reduction for each $1< \alpha < 2$. To avoid disrupting the flow of the paper we have deferred the details of the L-reduction to the “Appendix”.

Note that one consequence of the L-reduction given in the proof of Theorem 4 is that if RUHN has a constant-factor polynomial-time approximation algorithm (i.e. is in APX), then so does HN. In [22] it is proven that, if HN is in APX, so is Directed Feedback Vertex Set. Hence the following corollary is obtained.

#### Corollary 2

If RUHN is in APX, then so is HN and thus also Directed Feedback Vertex Set.

Determining whether Directed Feedback Vertex Set is in APX is a longstanding open problem in computer science; the corollary can thus be viewed *provisionally* as a strengthening of Theorem 4.

### Parameterized Complexity of RUHN

In this subsection we will show that RUHN is FPT when the parameter is $h^{ru}(\mathcal {T})$ (or, in other words, the size *k* of the optimal solution for RUHN). To prove this, we will provide a kernel of quadratic size which (when combined with any exponential-time algorithm) will let us answer the question “*Is the optimal solution to* RUHN $\le k$?” in time $O(f(k) \cdot \text {poly}(n))$ for some computable function *f* that depends only on *k*.

For the kernelization proof we use the same ingredients introduced in Sect. 3.2 and in particular the two reductions rules introduced there: **Common Pendant Subtree** (CPS) reduction and **Common** *d*-**Chain** (*d*-CC) reduction rules. We use them slightly differently from how they were defined there, because here the input to each reduction rule is a set of unrooted binary trees, and within the common chain reduction we will take $d = 5k$ (i.e., long common chains will be truncated down to length 5*k*). In [32] the authors described how these two reduction rules can be used in the *rooted* HN problem to reduce the initial instance ${\mathcal {T}}$ to a new kernelized instance of rooted binary phylogenetic trees $\mathcal {T}'$ on a set of leaf labels $X'$ such that $h^r(\mathcal {T}) \le k \Leftrightarrow h^r(\mathcal {T}') \le k$ and, moreover, $|X'| = O(k^2)$. Here, we adapt their arguments to work for the unrooted case as well. Although this might seem a direct generalization, additional technicalities must be addressed arising in root placement on the unrooted trees/networks.

We start by defining the concept of *generator* [21] which will be used in the rest of the section: An *r*-*reticulation generator* (for short, *r*-generator) is defined to be a directed acyclic multigraph with a single node of indegree 0 and outdegree 1, precisely *r* reticulation nodes (indegree 2 and outdegree at most 1), and apart from that only nodes of indegree 1 and outdegree 2. The *sides* of an *r*-generator are defined as the union of its edges (the edge sides) and its nodes of indegree-2 and outdegree-0 (the node sides). Adding a set of labels *L* to an edge side (*u*, *v*) of an *r*-generator involves subdividing (*u*, *v*) to a path of |*L*| internal nodes and, for each such internal node *w*, adding a new leaf $w'$, an edge $(w, w')$, and labeling $w'$ with some taxon from *L* (such that *L* bijectively labels the new leaves). On the other hand, adding a label *l* to a node side *v* consists of adding a new leaf *y*, an edge (*v*, *y*) and labeling *y* with *l*. In [32] it was shown that if *G* is an *r*-generator, then *G* has at most $4r-1$ edge sides and at most *r* node sides.

#### Theorem 5

Let $\mathcal {T}$ be a collection of binary, unrooted, phylogenetic trees on a common set of leaf labels (taxa) *X*. Let $\mathcal {T}'$ be the set of binary, unrooted, phylogenetic trees on $X'$ after we have applied the common pendant subtree (CPS) and the common chain (5*k*-CC) reduction rules, until no such rule can be performed anymore. Then $h^{ru}(\mathcal {T}) \le k \Leftrightarrow h^{ru}(\mathcal {T'}) \le k$ and, moreover, $|X'| = O(k^2)$.

We will start by showing that the (CPS) reduction rule leaves the hybridization number unchanged:

#### Claim 1

Let $\mathcal {T}$ be a set of unrooted binary trees with leaves labeled bijectively by *X*. Let *T* be a maximal common pendant subtree of $\mathcal {T}$ and let $\mathcal {T}'$ be the set of all trees in $\mathcal {T}$ after the application of the (CPS) reduction rule to *T*. Then $h^{ru}(\mathcal {T}) \le k \Leftrightarrow h^{ru}(\mathcal {T'}) \le k$.

#### Proof

Let *N* be the optimal (with the minimum reticulation number) network that displays the optimally rooted version of $\mathcal {T}$ and let $N'$ be the optimal network that displays the optimally rooted reduced instance $\mathcal {T}'$ (after a single application of the (CPS) reduction rule).

($\Leftarrow$) Let $h^{ru}(\mathcal {T}') = r(N') = k$. From $N'$ we will construct a rooted network *N* with *k* reticulation nodes that displays $\mathcal {T}$. Since $N'$ displays $\mathcal {T}'$ which is a collection of trees with leaves bijectively labeled from $\{X {\setminus } \{ X_T\}\} \cup \{x\}$ (where, as before, $X_T$ is the set of taxa of *T*), simply replace on $N'$ the leaf *x* with the common pendant subtree *T*. We have a new network $N''$ whose reticulation number obviously is *k* (we did not add/create any new reticulation nodes). The leaves of $N''$ are labeled from *X* (without *x*). It remains only to show that $N''$ displays $\mathcal {T}$ which is immediate since *T* displays itself. Observe that the root placement on each tree $T \in \mathcal {T}'$ is irrelevant.

($\Rightarrow$) For the other direction, consider $\mathcal {T}$ and let $\mathcal {T}^{\rho }$ be a rooting of all trees such that $h^{r}(\mathcal {T}^{\rho })$ is minimized. Let *N* be the rooted network displaying the trees in $\mathcal {T}^{\rho }$ and let $\rho (T)$, for $T \in \mathcal {T}$ be the actual root of *T* (given by $\mathcal {T}^{\rho }$). Similar for *N*. Let *T* be the CPS of each member of $\mathcal {T}$. From *N* we need to construct a new network $N'$ with *k* reticulation nodes that displays all the trees in $\mathcal {T}'$. The problem will be: what if $\exists T \in \mathcal {T}$ such that its root is inside *T*? In such cases, the (CPS) reduction rule will cut-off the root of this tree and this will “force” us to root *T* in another location unaffected by the (CPS) reduction rule, which will potentially change the hybridization number of the resulting instance. Given a rooting of all members of $\mathcal {T}$ and *N* with $h^{ru}(\mathcal {T}) = k$, consider the following rootings for each $T' \in \mathcal {T}'$: if $\rho (T) \in T$ then root $T'$ (after the clipping of *T*) on the parent of *x* (the new taxon replacing *T*). Else, leave the rooting unchanged. Now, from *N*, we need to create a new rooted network $N'$ that displays $\mathcal {T}'$ such that its reticulation number is (not greater than) *k*. Apply the standard procedure: let $X_T \subset X$ be the set of leaves of the CPS *T* and let $(x_1, x_2, \ldots , x_t)$ be some arbitrary but fixed ordering of them. Start with $x_1$, delete it and delete any reticulation node with outdegree 0 and perform the standard cleaning-up operations^2^ until the resulting network is a phylogenetic network. Repeat for $x_2$ and so on until arriving at $x_t$ which is simply relabelled by the new taxon *x*. Let $N'$ be the resulting network. By construction, $N'$ displays all $T_i' \in \mathcal {T}'$ and $r(N') \le k$.

Now to the common chain reduction rule:

#### Claim 2

Let $\mathcal {T}$ be a set of binary, unrooted trees on *X* and let $\mathcal {T}'$ be the set of trees in $\mathcal {T}$ after a single application of the (5*k*-CC) reduction rule. Then, $h^{ru}(\mathcal {T}) \le k \Leftrightarrow h^{ru}(\mathcal {T}') \le k$.

#### Proof

For the first direction (from the reduced to the original instance) let $C = (x_1, x_2, \ldots , x_t )$ be a subset of the taxa *X* that defines a maximal common chain of length $> 5k$. Suppose that, in $\mathcal {T}$, we have clipped *C* down to a reduced chain $C_R = (x_1,\ldots ,x_{5k})$. Let $\mathcal {T}_R$ be the set of these clipped (or reduced) trees and let $N_R$ be a network that displays some rooted version of $\mathcal {T}_R$ with *k* reticulation nodes. Since the generator has at most $5k-1$ sides, there must exist at least one side containing at least two leaves of the chain. Let $x_i$ and $x_j$ be two leaves of the chain that are on the same side *s* of the generator, with $x_i$ above $x_j$. Clearly, this side must be an edge side. We will consider the case that $i<j$. The case that $j<i$ can be handled symmetrically.

First suppose that $\{i,j\}\ne \{1,2\}$. Then, we move all the taxa of the chain on the appropriate location on the side *s* of $x_i,x_j$ of the generator *G*. We take all taxa $x_{\ell }$ such that $\ell > j$ and plug them after $x_j$ in *s*, by appropriately subdividing the unique edge exiting the parent of $x_j$. We do the opposite for all the taxa $x_{\ell '}$ such that $\ell ' < i$ i.e., plug them “before” $x_i$ in *s* by appropriately subdividing the unique edge entering the parent of $x_i$.

Now suppose that $i=1$ and $j=2$. Then we take any other pair of leaves that are on the same side of the generator and go back to the previous case. To see that such leaves exist, assume that $\{x_1,x_2\}$ is the only pair of leaves that are on the same side. If the trees in $\mathcal {T}_R$ are not all identical, then there exists at least one leaf *y* that is not in the reduced chain $C_R$. Since the generator has at most $5k-1$ sides, and the chain has 5*k* leaves, this implies that each side contains at least one leaf of the chain. Let $x_q$ be a leaf of the chain that is on the same side as *y*. This is only possible when $q\in \{1,5k\}$. If $q=1$ this implies that the original chain *C* was not maximal and we obtain a contradiction. If $q=5k$, then we can add *y* to $C_R$ and obtain a longer common chain $C_R'$. Repeating this argument, we eventually obtain a contradiction or find out that all trees in $\mathcal {T}_R$ are identical (a case that can be handled trivially).

As mentioned before, the case that $j<i$ can be handled symmetrically. In this case, we make sure that $\{i,j\}\ne \{5k-1,5k\}$.

*Expanding Step* We still need to expand the chain by introducing the “missing” taxa (the ones that disappeared after the clipping of the chain). Move all these taxa $\{ x_{5k+1}, \ldots , x_{t} \}$ to the side *s* in such a way that either *C* or the reverse sequence becomes a chain in the network. In that way, from $N_R$ on $X_R$ (the leaf label set without the clipped labels after an application of the (5*k*-CC) rule) we have created a new network *N* on *X* with the same reticulation number as $N_R$. We still need to argue that *N* displays some appropriately rooted version of $\mathcal {T}$.

Take any tree $T_R \in \mathcal {T}_R$. Perform all the previous operations (applied on $N_R$) on $T_R$. In other words, move appropriately all the corresponding taxa on the same side of the root and re-introduce the “missing” taxa in such a way that either *C* or the reverse sequence becomes a chain in the tree. In this way, from the rooted network $N_R$ on $X_R$ that displays a rooted version of the truncated trees in $\mathcal {T}_R$, we construct a new rooted network *N* on *X* *and* rooted versions $\mathcal {T}^{\rho }$ of the trees $\mathcal {T}$.

We now argue that *N* displays $\mathcal {T}^{\rho }$. Let $T^\rho \in \mathcal {T}^{\rho }$, let $T_R$ be the corresponding reduced tree in $\mathcal {T}_R$ and let $T_R^\rho$ be a rooting of the reduced tree $T_R$ that is displayed by $N_R$.

If neither $x_1$ and $x_2$ nor $x_{5k-1}$ and $x_{5k}$ have a common parent in $T_R^\rho$, then it is clear from the construction that *N* displays $T^\rho$. If $x_1$ and $x_2$ have a common parent in $T_R^\rho$, then it is possible that $x_1$ and $x_2$ are on the same side of the generator of $N_R$ with $x_1$ above $x_2$. If we moved all chain-taxa below $x_2$ on this side then this would invert the chain, which would be a problem. However, this does not happen since in this case $i<j$ and in that case we made sure that $\{i,j\}\ne \{1,2\}$. Symmetrically, if $x_{5k-1}$ and $x_{5k}$ have a common parent in $T_R^\rho$ and $x_{5k}$ is above $x_{5k-1}$ on some side of $N_R$, then we do not move all taxa to this side because then $j<i$ and hence $\{i,j\}\ne \{5k,5k-1\}$.

For the other direction (from the original instance to the truncated), let *N* be a rooted network that displays some rooted version of $\mathcal {T}$ with *k* reticulation nodes. Let $\mathcal {T}_R$ be the set of trees from $\mathcal {T}$ after a single application of the common chain reduction rule. Then, from *N*, we will show how to create a rooted network $N'$ that displays some appropriately rooted version of $\mathcal {T}'$. Let $N'$ be the network obtained from *N* as follows: let *C* be a common chain on $\mathcal {T}$ of length greater than 5*k*. Take *C* on *N* and “clip” it i.e., delete all leaves $x_\ell$ with indexes $\ell \ge 5k+1$, and apply the usual cleaning-up steps. If the root of *N* happens to be on the chain then take the single edge *e* entering the parent of the last surviving taxon of the chain with index $x_{5k}$, subdivide it and introduce the new root location at the new intermediate node that subdivides *e*. Do the same on all $T \in \mathcal {T}$. Thus, we have created a new rooted network $N'$ and a rooting for all trees in $\mathcal {T}_R$, all on $X'$ (without the “excess” taxa deleted from the common chain). Obviously, by construction, the reticulation number of $N'$ has not increased. It remains to show that $N'$ displays $\mathcal {T}_R$ which follows immediately since *N* displays (a rooted version of) $\mathcal {T}$.

These two claims show that successive applications of the (CPS) and (5*k*-CC) rules do not change the hybridization number of the resulting reduced instances. Assuming that we have applied these two rules as often as possible, let $\mathcal {T}'$ be the resulting instance. From the previous analysis we know that $\exists N'$ such that $r(N') \le k$. Since each common chain of $\mathcal {T}'$ has length $\le 5k$, we conclude that in $N'$ we cannot find a chain of length greater than 5*k* where all leaves are on the same side of the underlying generator (otherwise it would constitute a common chain and it would be clipped). Thus, $N'$ has at most $5k-1$ taxa on each *edge* side and, obviously, at most one taxon on each *node* side. Thus, the total number of taxa that $N'$ can have is at most

$$\begin{aligned} (5k-1) \cdot \underbrace{(4k-1)}_{\# \text { of edge sides}} + \underbrace{k}_{\# \text { of node sides}} < 20k^2. \end{aligned}$$

The above kernelization eventually terminates: at each step we either identify a common pendant subtree or a long common chain or, if none of these is possible, we terminate. Each reduction step reduces the number of taxa by at least 1, so we eventually terminate in polynomial time.

The kernel we have described can be used to give an FPT algorithm to answer the question, “Is $h^{ru}(\mathcal {T}) \le k$?”. Let $\mathcal {T'}$ be the kernelized set of trees. If the cardinality of the set of leaves given in the above bound is violated, we know that the answer is NO. So, assume it is not violated. We simply guess by brute-force the root location of each tree in $\mathcal {T'}$. Each collection of guesses yields a set of rooted binary phylogenetic trees $\mathcal {T''}$, and we ask “Is $h^{r}(\mathcal {T''}) \le k$?” Clearly, the answer to “Is $h^{ru}(\mathcal {T}) \le k$?” is YES if and only if at least one of the “Is $h^{r}(\mathcal {T''}) \le k$?” queries answers YES. The kernelization procedure ensures that each tree in $\mathcal {T'}$ has $O(k^2)$ taxa and thus also $O(k^2)$ edges. Hence, the overall running time is the time for the kernelization procedure plus $[O(k^2)]^t$ calls to an algorithm for HN, where $t = |\mathcal {T}|$. Noting that $t \le 2^k$ (otherwise the answer is trivially NO), and that HN is FPT [31], we obtain an overall running time of $O( \text {poly}(n) + f(k) \cdot \text {poly}(n) ) = O(f(k) \cdot \text {poly}(n))$.

This concludes the proof of Theorem 5.

## Conclusions and Open Problems

In this article we have studied two variations of the classical hybridization number (HN) problem: the root-uncertain variant RUHN and the unrooted variant UHN. We have also studied the natural unrooted variant of the tree containment (TC) decision problem, UTC.

As we have seen, both TC and UTC are NP-complete and FPT in reticulation number. The natural open question here is whether our FPT algorithm for UTC, with running time $O( 4^k \cdot \text {poly}(n) )$, can be improved to achieve a running time of $O( 2^k \cdot \text {poly}(n) )$, which is trivial for TC. Also, the TC literature has not yet considered pre-processing, so it would be interesting to adapt our kernelization strategy to the rooted context.

Regarding HN and RUHN, both are APX-hard. It is known that if HN (respectively, RUHN) is in APX then so too is DFVS. However, at present we do not have a reduction from RUHN to HN, which means that (from an approximation perspective) HN might be easier than RUHN. This is an interesting question for future research: it remains a possibility that both HN and DFVS are in APX, but RUHN is not. Both HN and RUHN are FPT in hybridization number, via a quadratic kernel. For RUHN a pertinent question is whether, in the case of just *two* input trees, the best known FPT running time for HN can be matched, which is $O(3.18^{k} \cdot \text {poly}(n))$ [35]. This raises the question of whether, and in how far, the successful agreement forest abstraction can be adapted for RUHN.

In terms of approximation the other variant of HN, UHN, differs quite strikingly from HN, although we note that in this article we have only studied UHN on two trees. For two trees RUHN is (due to its equivalence with TBR) in APX, while it is still unknown whether HN is in APX (see the above discussion). This gap in approximability is similar to that which exists between Maximum Acylic Agremeent Forest (MAAF) and Maximum Agreement Forest (MAF) on two rooted trees [22]. This is not so surprising given that MAAF is essentially equivalent to HN, and both the rooted and unrooted variants of MAF (which are essentially equvalent to rSPR and TBR respectively) are firmly in APX.

Alongside the complexity discussions above it is tempting to ask which of the problems studied in this article can (in some formal sense) be “reduced” to each other. The APX-hardness reduction already shows that HN can be reduced to RUHN in a highly approximation-preserving way. Can RUHN be reduced to HN? Can HN be reduced to UHN? Can RUHN be reduced to UHN?

## Omitted APX-Hardness Proof

Here we give the details of the omitted proof that RUHN is APX-hard (Theorem 4).

### Proof

To establish the result we provide a linear (L) reduction [25] from HN to RUHN. Formally, an L-reduction is defined as follows:

### Definition A.1

Let *A*, *B* be two optimization problems and $c_A$ and $c_B$ their respective cost functions. A pair of functions *f*, *g*, both computable in polynomial time, constitute an L-reduction from *A* to *B* if the following conditions are true:

1. *x* is any instance of *A* $\Rightarrow$ *f*(*x*) is an instance of *B*,
2. *y* is a feasible solution of *f*(*x*) $\Rightarrow$ *g*(*y*) is a feasible solution of *x*,
3. $$\exists \alpha \ge 0$$ such that $$OPT_B(f(x)) \le \alpha OPT_A(x)$$,
4. $\exists \beta \ge 0$ such that for every feasible solution $y'$ for *f*(*x*) we have $|OPT_A(x) - c_A(g(y'))| \le \beta |OPT_B(f(x)) - c_B(y')|$

where $OPT_{A}$ is the optimal solution value of problem *A* and similarly for *B*.

HN is already known to be NP-hard and APX-hard for $|\mathcal {T}|=2$. We will give a $(\alpha , \beta )=(2,1)$ L-reduction from HN to RUHN.

Let $(T_1, T_2)$ be the two trees that are given as input to HN, and let *X* be their set of taxa, where $n=|X|$. Let $(T^{*}_1, T^{*}_2)$ be the unrooted trees constructed in the proof of Lemma 4, except that here we make the “*c*” and “*d*” caterpillars slightly longer: length $(2n+3)$ instead of length $(n+1)$. It is easy to check that with these longer caterpillars (2) still holds.

To avoid technical complications (the approximation oracle outputting an exponentially large answer) it is helpful in this proof to assume that, when given two unrooted binary trees as input, each on $n'$ taxa, an approximation oracle for RUHN never returns a network *N* such that $r(N) > n'$. This is reasonable because one can always root the two trees in arbitrary places and construct a trivial network with $n'$ reticulation nodes that displays both rootings, simply by “merging” the two rooted trees at their taxa and adding a new root.

The reduction proceeds as follows. First we check whether $h^{r}( T_1,T_2 ) = 0$. This can easily be performed in polynomial time since this holds if and only if $T_1$ and $T_2$ are isomorphic. If the equality holds, then there is no need to call the RUHN approximation oracle: simply return $T_1$ as an optimal solution to HN (i.e. $T_1$ is a rooted phylogenetic network that trivially displays both $T_1$ and $T_2$). Otherwise, we know $h^{r}(T_1, T_2) \ge 1$. Given that (as shown in the previous proof) $h^{ru}(T^{*}_1,T^{*}_2 ) = h^{r}(T_1, T_2) + 1$ it then follows immediately that $h^{ru}(T^{*}_1,T^{*}_2 ) \le \alpha \cdot h^{r}(T_1, T_2)$, thus satisfying the “forward mapping” part of the L-reduction (i.e. point 3 of the definition).

We now consider the “back mapping” part, i.e. point 4 of the definition. Consider the solution returned by the approximation oracle for RUHN. We distinguish two cases. First, suppose the solution given by the oracle roots $T^{*}_1$ and $T^{*}_2$ in a “stupid” way i.e. such that two oppositely oriented rooted caterpillars are induced. If this happens, the network given by the oracle will have reticulation number at least $2n+1$ (i.e. the length of the caterpillar minus two). We know $h^{ru}(T^{*}_1,T^{*}_2 ) = h^{r}( T_1,T_2 ) + 1$ so, trivially, $h^{ru}(T^{*}_1,T^{*}_2 ) \le n+1$. Hence, the additive gap between the quality of the approximate and exact solution to $\textsc {RUHN}(T^{*}_1,T^{*}_2)$ is at least $(2n+1)-(n+1) = n$. To complete the L-reduction in this case, we simply discard the output of the approximation oracle, and return a trivial solution to $\textsc {HN}$ with *n* reticulations (e.g. by merging $T_1$ and $T_2$ at their taxa and adding a new root). Given that $h^{r}(T_1,T_2 ) \ge 1$, the additive gap between our approximate solution to $\textsc {HN}$ and its true optimum is at most $n-1$. Clearly, $n-1 \le \beta \cdot n$, as required by the “back mapping” part of the L-reduction.

The other case is if the the approximate solution roots the trees in a “sensible” way. That is, without loss of generality, $T^{*}_1$ is rooted somewhere inside its *c*-part and $T^{*}_2$ is rooted somewhere above its $T_2$ part (i.e. somewhere in its *c*-part or *d*-part). This case is somewhat more subtle. Suppose the network $N'$ returned by the approximation oracle for RUHN has reticulation number *q*.

We restrict $N'$ to only those nodes and edges necessary to display $T_1$ and $T_2$. The images of $T_1$ and $T_2$ inside $N'$ can easily be found in polynomial time because (a) images of the rootings of $T^{*}_1$ and $T^{*}_2$ within $N'$ will be returned by the oracle as certificates, and (b) due to the location of the chosen roots, images of $T_1$ and $T_2$ within $N'$ can be directly extracted from the images of the rootings of $T^{*}_1$ and $T^{*}_2$. (Note that if $N^{T_1, T_2}$, which simply denotes the union of the two images, does not comply with the degree restrictions of a rooted binary phylogenetic network, it is easy to modify it so that it does comply with these restrictions, without raising its reticulation number). $N^{T_1, T_2}$ displays $T_1$ and $T_2$, and has *X* as its set of taxa: this will be the solution we return for HN.

Suppose that $N^{T_1, T_2}$ has reticulation number *p*. Given that the restriction operation does not create new reticulation nodes (but possibly deletes them), $p \le q$. To make the reduction go through, we need:

$$\begin{aligned} |p - h^{r}(T_1,T_2)| \le \beta | q - h^r(T^{*}_1, T^{*}_2) | \end{aligned}$$

which is equivalent to,

$$\begin{aligned} |p - h^{r}(T_1, T_2)| \le | q - h^{r}(T_1, T_2) - 1 | \end{aligned}$$

If $p \le (q-1)$ this is immediate. However, what if $p=q$? This occurs if $N^{T_1, T_2}$ has just as many reticulations as $N'$ i.e. the restriction process did not delete any reticulation nodes. However, this cannot happen, for the following reason. Consider again the image of the rooting of $T^{*}_1$ in $N'$. Extract all nodes and edges on the image that minimally span the taxa $\{c_0, \ldots , c_{2n+2}, d_0, \ldots , d_{2n+2}\}$. Do this also for the image of the rooting of $T^{*}_2$ in $N'$. Let $N^{Cat}$ be the network obtained from $N'$ by restricting to the union of these nodes and edges. $N^{Cat}$ is a rooted binary phylogenetic network on $\{c_0, \ldots , c_{2n+2}, d_0, \ldots , d_{2n+2}\}$ that displays the caterpillars induced by the rootings of $T^{*}_1$ and $T^{*}_2$. Crucially, $r(N^{Cat}) > 0$. This is because, as observed earlier, the caterpillar parts of $T^{*}_1$ and $T^{*}_2$ remain non-isomorphic wherever you place the root exactly. In turn this means that $N^{Cat}$ must contain at least one reticulation node *v*. This reticulation node *v* necessarily lies on the images of both $T^{*}_1$ and $T^{*}_2$, since otherwise it would not have survived to be a reticulation node in $N^{Cat}$ (i.e. at most one of its two incoming edges would have been extracted, and hence it would have been suppressed). However, *v* does not lie on the part of the image of $T^{*}_1$ that contributed to $N^{T_1, T_2}$, since *v* is not part of the image of $T_1$. Hence, *v* lies on (at most) the image of $T_2$, meaning that *v* is a reticulation node that does not survive when $N^{T_1, T_2}$ is created (because at most one of its two incoming edges is extracted), and hence $p < q$. $\square$

The above proof can, if desired, be strengthened to an $(\alpha , 1)$ L-reduction for any constant $1< \alpha < 2$. To achieve this, we do not begin by checking whether $h(T_1, T_2)=0$, but instead whether $h(T_1, T_2) < \frac{1}{\alpha -1}$, and if so we simply return an optimal solution. For constant $\alpha$ this can be performed in polynomial time, because computation of HN is FPT in $h^{r}(T_1, T_2)$.

## Footnotes

1 This follows from [26]. In that article the hardness of NDP is proven for undirected graphs of maximum degree 3, but using standard gadgets nodes of degree 1 or 2 can easily be upgraded to degree 3. See also [28], p. 1225 for a related discussion.

2 Deleting reticulation nodes with outdegree 0; suppressing nodes with indegree and outdegree both equal to 1; deleting leaves unlabelled by taxa; deleting nodes with indegree 0 and outdegree 1.
